# Heterozygous GAA knockout is nonconsequential on metabolism and the spatial liver transcriptome in high‐fat diet‐induced obese and prediabetic mice

**DOI:** 10.14814/phy2.70276

**Published:** 2025-03-19

**Authors:** Cameron P. McCall, Melina C. Mancini, Jaroslaw Staszkiewicz, Douglas G. Mashek, Timothy D. Heden

**Affiliations:** ^1^ Pennington Biomedical Research Center Baton Rouge Louisiana USA; ^2^ Department of Biochemistry, Molecular Biology, and Biophysics University of Minnesota Saint Paul Minnesota USA; ^3^ Department of Medicine, Division of Diabetes, Endocrinology, and Metabolism University of Minnesota Saint Paul Minnesota USA

**Keywords:** glycogen autophagy, glycophagy, liver, metabolism, prediabetes

## Abstract

Glycophagy is the autophagic degradation of glycogen by the enzyme acid alpha‐glucosidase (GAA). Although GAA inhibitors improve metabolic health by inhibiting GAA in the intestine, it is not clear if GAA inhibition in peripheral tissues such as the liver is metabolically beneficial. This study tested if the heterozygous knockout of GAA (HetKO‐GAA) alters liver metabolism and metabolic health in mice fed a low‐fat diet or a high‐fat diet to induce obesity. HetKO‐GAA mice fed either diet did not have altered body weight, glucose tolerance, insulin action, energy expenditure, substrate metabolism, liver glucose output, or liver triglycerides compared to control wildtype mice. A liver spatial transcriptomics analysis revealed that high‐fat diet feeding reduced the gene abundance of predominantly metabolic pathways in both periportal and perivenous hepatocytes, and uniquely reduced ribosome gene abundance in perivenous hepatocytes. HetKO‐GAA mice did not have significantly altered transcriptomes in periportal or perivenous hepatocytes compared to wildtype mice. In conclusion, heterozygous GAA knockout is nonconsequential on metabolism and metabolic health in high‐fat diet induced obesity. Spatial transcriptomics revealed alterations in the transcriptome of periportal and perivenous hepatocytes from high‐fat diet induced obese mice, highlighting novel targets that could be exploited to improve metabolic health in obesity.

## 1 INTRODUCTION

Obesity and prediabetes are major global health problems that increase the risk of developing metabolic associated fatty liver disease (MAFLD), type 2 diabetes, and heart disease, with the latter two being leading causes of death (Lim & Boster, 2024). Despite an expanding armamentarium for these conditions, side effects and medical expenses represent significant obstacles to treatment. With obesity affecting over 1 billion people (Collaboration NCDRF, 2024) and prediabetes affecting 464 million people (Collaborators GBDD, 2023; Rooney et al., 2023) worldwide, there is still a considerable need for exploration of novel metabolic pathways that could be used as targets to prevent the advance of the obese, MAFLD, and prediabetic states to type 2 diabetes.

Glycogenolysis is a central metabolic pathway in metabolism. Glycogenolysis occurs through two different cellular compartments, including in the cytosol via the enzymes glycogen phosphorylase and debranching enzyme, or through autophagy in a process referred to as glycophagy, where the lysosomal enzyme acid alpha‐glucosidase (GAA) degrades glycogen to glucose. FDA‐approved GAA inhibitors, such as acarbose or miglitol, are effective at improving insulin action and glycemic control while also being effective at reducing fatty liver in individuals with obesity and type 2 diabetes (Goda et al., 2007; Joubert et al., 1990; Komatsu et al., 2018; Nozaki et al., 2009). These drugs are thought to prevent the digestion and absorption of complex carbohydrates in the intestine (Ahr et al., 1989; Joubert et al., 1990). It is less understood if GAA inhibition in peripheral tissues has metabolic health benefits. Glycogen storage disease type II, or Pompe disease, is a genetic disorder where GAA enzyme activity is deficient and autolysosomal (autophagosome and lysosome) glycogen accumulates (Bay et al., 2019). In mice, there is evidence that GAA deficiency (i.e., Pompe disease) increases glucose tolerance compared to wildtype (Wt) littermates (Douillard‐Guilloux et al., 2010). Furthermore, there is evidence that glycophagy‐derived glucose may be directed toward liver glucose output (Douillard‐Guilloux et al., 2010; Hijmans et al., 2017), which is relevant for diabetes as liver glucose output is typically upregulated and dysregulated in diabetes (Chevalier et al., 2006). We and others have shown that glycophagy deficiency in skeletal muscle augments insulin action in mouse muscle cells (Heden et al., 2022) or insulin signaling in mouse muscle (Lim et al., 2017). However, a detailed characterization of the effects of GAA deficiency on metabolic health has not been performed. Therefore, the objective of this study was to determine how heterozygous knockout of GAA (HetKO‐GAA) in mice impacts metabolic health (i.e., body weight, insulin sensitivity, glucose tolerance, fatty liver) and metabolism (i.e., liver glycogen content, liver glucose output, substrate metabolism, energy expenditure, liver transcriptome) in a mouse model of high‐fat diet‐induced obesity and prediabetes. We find that HetKO‐GAA is nonconsequential on all indices of metabolic health assessed. Spatial transcriptomics in the liver revealed that high‐fat diet‐induced obesity and prediabetes robustly reduced mitochondrial metabolism‐related gene abundance in both periportal and perivenous hepatocytes and uniquely reduced ribosome gene abundance in perivenous hepatocytes.

## 2 MATERIALS AND METHODS

### 2.1 Mouse models and genotyping

The University of Minnesota Mouse Genetics Lab generated the heterozygous knockout of GAA (HetKO‐GAA) and homozygous knockout of GAA (KO‐GAA) mice used in this study. Briefly, the Tild‐CRISPR/Cas9 system (Yao et al., 2018) was used to knock in LoxP sites so that they were flanking Exon 4 and 5 of the GAA gene. The LoxP site incorporation efficiency by TILD at the GAA locus intron 3 and intron 5 was confirmed in NIH3T3 cells (data not shown). The plasmid was then microinjected into 605 zygotes, of which 403 survived while 270 were transferred into females. Of the 21 pups born, 12 had LoxP knock‐in, and 2 had LoxP knock‐in on the GAA locus (founder mice). After confirming germline transmission and that LoxP was inserted on the GAA locus, mice with heterozygous knock‐in of LoxP were backcrossed to C57BL/6 mice, and then mice with heterozygous LoxP knock‐in were bred together to produce the male and female mice for this study. The effects of the Tild‐CRISPR/Cas9 system and LoxP sites (Chehelgerdi et al., 2024; Simkin et al., 2022; Skryabin et al., 2020; Suchy et al., 2024; Wang et al., 2021; Yen et al., 2014) on the GAA gene constitutively disrupted GAA transcription and GAA activity from birth to produce whole body HetKO‐GAA or KO‐GAA mice. The forward primer (5' to 3') CAT TGG GTC CCA GAG GTT CC and the reverse primer (5' to 3') AGG AAC TGG TCA GCG AAG AA were used for genotyping and distinguishing between Wt, HetKO‐GAA, or KO‐GAA mice. The restriction enzyme PsiI‐v2 (New England Biolabs, R0744L) was used to digest the PCR product to make the Wt, HetKO‐GAA, and KO‐GAA bands easier to distinguisable. The db/db mouse line was purchased from The Jackson Laboratory (Strain # 000697).

### 2.2 Mouse study

All studies performed in this manuscript were approved by the University of Minnesota IACUC or the Pennington Biomedical Research Center IACUC. All mice were housed at ~22–23°C with a 12‐h light and 12‐h dark cycle. The male and female db/+ and db/db mice were sacrificed, and liver tissue was collected at ~10 weeks of age. For the diet study, male and female mice approximately 8 weeks of age were ad libitum fed a high‐fat diet (HFD, TD.06415, Inotiv) or low‐fat diet (LFD, TD.180916, Inotiv) for 15 weeks and then sacrificed. The mice were weighed every week for the duration of this study. Tolerance tests were conducted weeks 7–11. NMR, body composition, and indirect calorimetry were conducted weeks 10–14. Mice were sacrificed 15 weeks after starting their respective diet. The method of euthanasia included administering a cocktail of ketamine (120 mg per kg of body weight), xylazine (9 mg per kg of body weight), and acepromazine (2 mg per kg of body weight) via an intraperitoneal injection, and once the mice were anesthetized, a cardiac stick for blood collection was performed, the mice were cervically dislocated, and then the heart was removed. Tissue samples were weighed, flash‐frozen in liquid nitrogen, and stored at −80°C. Blood plasma was isolated and stored at −80°C. Histological sections were preserved in 10% formalin.

### 2.3 Glucose, insulin, and glycerol tolerance tests

Blood glucose was measured with a glucometer from a tail snip at baseline and every 15 min after administration of glucose (Millipore Sigma, 49,139), glycerol (Millipore Sigma, 49,767), or insulin (Lilly, Humulin®R). For the glucose tolerance test, mice were fasted for 3.5 h and then challenged with an oral gavage of 3 mg of glucose per gram of body weight. For the insulin tolerance test, mice were fasted for 2 h and then challenged with an intraperitoneal (I.P.) injection of 1.5 U of insulin per kg of body weight for males or 1 U of insulin per kg of body weight for females. For the glycerol tolerance test, mice were fasted for 5 h and then challenged with an I.P. injection of 1 mg of glycerol per gram of body weight. Mice were re‐fed upon completion of all tests.

### 2.4 Primary hepatocyte isolation

Mice between 8 and 10 weeks of age were anesthetized, and primary hepatocytes were isolated as previously described (Charni‐Natan & Goldstein, 2020). Cells were counted with 1:1 dilution trypan blue and then plated for insulin action or hepatocyte glucose output experiments (described below). An aliquot of hepatocytes was spun down, flash‐frozen in liquid nitrogen, stored at −80°C, and used to validate the heterozygous knockout of GAA.

### 2.5 Insulin action

Three hours after plating hepatocytes, cells were serum‐starved in media (Thermo Fisher, 10,566,016) for 2.5 h, with or without 150 μM sodium oleate (TCI, O0057) and 150 μM sodium palmitate (Millipore Sigma, P9767) added to the media for lipid loading (300 μM total lipid), prior to administering 100 nM of insulin (Millipore Sigma, I6634) for 10 min in serum‐starved media. Immediately after insulin stimulation, the cells were washed once with PBS (Thermo Fisher, 10,010,023) and the plates were frozen in liquid nitrogen and stored at −80°C. RIPA buffer (0.1% SDS [Millipore Sigma, 75,746], 0.001% Sodium deoxycholate [Millipore Sigma, 30,970], 0.1% Triton X‐100 (Millipore Sigma, T8787), 0.5 mM Tris–HCl (Millipore Sigma, 9310‐OP), 5 mM EDTA [Millipore Sigma, E9884], 0.15 M NaCl [Millipore Sigma, S9888], Protease [Pierce, A32963] & Phosphatase [Pierce A32957] inhibitor) was added to the wells, and the cell homogenate was transferred to a microcentrifuge tube for analysis of insulin signaling protein abundance using Western blot.

### 2.6 Hepatocyte glucose output

Primary hepatocytes were plated on three 12‐well 5 μg/cm^2^ rat tail collagen‐coated plates at 400,000 cells/well in 1 mL low glucose DMEM (Thermo Fisher, 10,567,014) with 5% FBS (Thermo Scientific, A5256701) added. Hepatocytes were incubated for 2 h at 37°C in 5% CO_2_. One plate was kept in the incubator to represent the “fed” state. The other two plates representing a “starved” state had their media removed and were washed three times with PBS (Thermo Fisher, 10,010,023); then 350 μL of minimal DMEM (Thermo Fisher, 1,443,001) without glucose, phenol red, or pyruvate was added to each well. For starved plates, half of the wells were treated with 100 nM glucagon (Millipore Sigma, G2044). Plates were incubated for 30 min at 37°C in 5% CO_2_ and 20 RPM. For all plates, media was harvested, flash‐frozen in liquid nitrogen, and stored at −80°C. The plates were then washed 2x with PBS (Thermo Fisher, 10,010,023), flash‐frozen in liquid nitrogen, and stored at −80°C. Glucose output into the media was measured with the FUJIFILM Wako Autokit Glucose (937–03001) and normalized to a glucose standard curve.

### 2.7 Liver glycogen, free glucose, and serum glucose

To measure glycogen in liver samples, the samples were first homogenized in 1 M HCl. Next, the sample was split into two tubes including one for measurement of cellular free glucose with the FUJIFILM Wako Autokit Glucose kit (937–03001), while the other sample was used for glycogen measurement and first heated at 95°C for 1 h to catalyze glycogen into constituent glucose, and glucose was measured with the FUJIFILM Wako Autokit Glucose kit (937–03001). Free glucose was substracted from glucose released from glycogen so that free glucose was not used as part of the glycogen measurement. Serum samples were assayed directly for glucose using the FUJIFILM Wako Autokit Glucose kit (937–03001).

### 2.8 GAA activity

Liver samples were homogenized in RIPA buffer, and absolute protein was measured with the Pierce BCA protein assay kit (Thermo Fisher, A55860) in 96‐well polystyrene plates using a BSA standard curve. GAA activity was measured using a previously described protocol (Heden et al., 2022). Briefly, 5 μL of sample was added to an acidic buffer containing 0.1 M citric acid (Millipore Sigma, C0759), 0.2 M disodium hydrogen phosphate (Millipore Sigma, 1.06585), 10 mM 4‐methyl‐umbelliferyl‐alpha‐glucopyranoside (Millipore Sigma, 69,591), pH 4.5, on a 96‐well black plate for 1 h at 37°C. After 1 h, the reaction was halted with the addition of a basic buffer containing 368 mM glycine (Millipore Sigma, G8898) and 445 mM sodium carbonate (Millipore Sigma, 223,530), pH 10.5. Additionally, 1 mM of the GAA inhibitor miglitol (TCI America, M23021G) was added to control samples to account for the background of the assay and to test if the assay was working properly. Fluorescence was read at 360/440 excitation/emission. GAA activity was normalized to absolute protein.

### 2.9 Liver and serum triglycerides and cholesterol

Liver samples were homogenized in ultrapure water (Apex Bioresearch Products 18–195), and lipids were extracted with 2:1 chloroform: methanol using the Folch method. The chloroform was evaporated under a dry nitrogen blower, and lipids were resuspended in isopropanol with 1% Triton‐X100 (Millipore Sigma, X100). Liver triglycerides were measured using a commercially available kit (Pointe Scientific, T75321L). Liver cholesterol was measured using a commercially available kit (Pointe Scientific, C7510120). Serum triglyceride and cholesterol levels were also measured using these kits.

### 2.10 Quantitative PCR (qPCR)

RNA was extracted from samples using Trizol (Thermo Fisher, 15,596,026). Concentration and purity were measured with a nanodrop. cDNA was synthesized via PCR with the Applied Biosystems High‐Capacity cDNA Reverse Transcription Kit (#4368814). PowerTrack™ SYBR™ Green Master Mix reagent was added to cDNA for the qPCR reaction mix, and qPCR was performed with the Applied Biosystems QuantStudio™ 6 Pro Real‐Time PCR System (#A43054) using Applied Biosystems Design & Analysis 2.6.0 software.

### 2.11 Western blotting

Samples were diluted in RIPA buffer. The initial run was performed at 120 V for 60 min, and the transfer step was performed at 100 V for 60 min. Protein loading was measured with a Ponceau S (Thermo Fisher, 161,470,250) stain. Rabbit AKT (Cell signaling 9272S) and pAKT^S473^ (Cell signaling 4060S) were used as primary antibodies, and fluorescent Goat anti‐rabbit (Li‐Cor IRDye® 800CW 926–32,211) was used as the secondary antibody.

### 2.12 Histology

Tissue sections were fixed in 10% formalin (Millipore Sigma, HT501128) and subsequently paraffin‐embedded for Hematoxylin and Eosin (H&E) and Periodic Acid Schiff (PAS) staining, which were performed within the Cell Biology and Bioimaging Core at Pennington Biomedical Research Center.

### 2.13 Spatial transcriptomics

#### 2.13.1 Liver preparation for NanoString GeoMx assay

Liver tissues from formalin‐fixed, paraffin‐embedded blocks were sectioned at a thickness of 5 microns and mounted on Superfrost Plus Microscope Slides (Fisherbrand, 12–550‐15). Sections were placed within the central 36.2 mm × 14.6 mm area of the slides to ensure tissues fit within the gasketed GeoMx slide holder. Mounted tissues were dried in a hood overnight at room temperature. Slides were processed and stained using the Nanostring GeoMx RNA slide preparation protocol. DEPC‐treated water (Thermo Fisher, AM9922) was used to make all solutions. Slides were baked for approximately 60 min at 60°C. After baking, tissue was deparaffinized and rehydrated with Citrisolv (Fisher Scientific, 04–355‐121), H_2_O, and EtOH. Antigen retrieval was performed with a high pH EDTA‐based antigen retrieval buffer (eBioscience IHC Antigen Retrieval Solution – High pH, 00–4956‐58). The antigen retrieval buffer was preheated for 1 h in a 5‐quart steamer (Hamilton Beach, 37530Z). Slides were incubated in hot EDTA buffer in the steamer for 20 min. To expose RNA targets for optimal probe hybridization, tissues were digested in a 1 μg/mL proteinase K (Invitrogen Ambion Recombinant Proteinase K Solution, AM2546) solution for 5 min at 37°C. Following proteinase digestion, tissues were briefly fixed in 10% neutral buffered formalin for 5 min.

#### 2.13.2 RNA hybridization and morphology marker staining

Prepared tissues were flooded with whole transcriptome RNA probes (NanoString Technologies, 121,401,102) attached to oligonucleotide barcodes via photocleavable linkers. Slides were covered with HybriSlip hybridization covers (Grace Bio‐Labs, 714,022) and hybridized overnight at 37°C in a hybridization oven (Boekel Scientific RapidFISH Slide Hybridizer, Cat#240200). Following hybridization, unbound probes were washed away using a solution of equal parts 4XSSC and deionized formamide (Thermo Fisher, AM9342). After washing, immunofluorescence staining was performed using commercially available antibodies against E‐Cadherin (BD Biosciences, Cat#610182) and Cytochrome P450 2E1 (Abcam, ab28146). Slides were blocked with Buffer W probes (NanoString Technologies, 121,300,313) at room temperature for 2 h incubated with unconjugated primary antibody overnight at 4°C and a concentration of 0.1667 μg/mL for E‐Cadherin and 20 μg/mL for Cytochrome P450 2E1. Slides were washed three times in 2XSSC and then incubated for 2 h with secondary antibodies conjugated with Alexa Fluor 532 and 647 (diluted 1:2000) and SYTO 13 green, fluorescent nucleic acid stain (Thermo Fisher, S7575). Slides were washed in 2XSSC and loaded into the GeoMx DSP instrument slide holder.

#### 2.13.3 GeoMx DSP area of illumination selection and barcode collection

The GeoMx DSP instrument performs multiplexed wide field immunofluorescence microscopy and utilizes an ultraviolet (UV) laser to release oligonucleotide barcodes from a specific area of illumination (AOI). The fluorescently labeled morphology markers enable precise selection of regions within a tissue based upon fluorescent intensity. The GeoMx instrument has a complement of four fluorescence imaging channels with the following specifications (Channel, Excitation (peak/bandwidth), Emission (peak/bandwidth)): FITC, 480/28 nm, 516/23 nm; Cy3, 538/19 nm, 564/15 nm; Texas Red, 588/19 nm, 623/30 nm; Cy5, 645/19 nm, 683/30 nm. Only the selected areas were illuminated by UV light, thereby photocleaving the linker molecule and liberating the oligonucleotide barcodes in specific regions within the AOI. Slides were loaded and scanned in the Nanostring GeoMx DSP instrument. Exposure times for each channel were optimized for each instrument run. Typical exposure times were set to 75 ms (FITC), 300 ms (Cy3), and 200 ms (Cy5) and modified as needed to optimize the fluorescence signal.

Each AOI was flooded with oligo‐labeled probes, each designed to hybridize with the mRNA from a specific gene. Each probe that successfully finds its mRNA target releases a short DNA fragment (oligo) that contains a sequence (or barcode) identifying the gene that the probe was designed to target. All these released DNA fragments from an AOI are collected and run on a sequencer to see what the DNA sequence is of each fragment. A lookup table provides the conversion from DNA sequence (or barcode) to gene target, and Nanostring software counts how many times the DNA barcodes for each gene were seen in the sequence data from a region of interest, resulting in a big count table. The count values indicate mRNA abundance, and these values were then normalized to the number of nuclei to correct for cell number in each AOI. Statistical analysis was performed on these corrected count numbers.

### 2.14 Metabolic and behavioral phenotyping

Mice were placed in Promethion Metabolic Cages (Hong Kong Polytechnic University) for 75 h. Oxygen consumption, energy expenditure, respiratory exchange ratio, activity, food intake, and water intake were measured using this closed system. The mice were fasted for approximately 17 h during the last day of testing.

### 2.15 Statistics and graph generation

GraphPad Prism 10.1.2 and CalR 1.3 (Abbassi‐Daloii et al., 2023) (https://www.calrapp.org/) were used to generate graphs and run analyses. Treatment groups were compared using Tukey multiple comparisons tests. DESeq2 v1.42.1 in R v4.3.3.R software was used to perform differential expression analysis of the spatial transcriptomics data set. Two criteria required for significance included an adjusted *p* value <0.05 as well as a Log2 Fold Change of ≥0.80 or ≤−0.8 or lower. Volcano plots were generated using VolcanoNoseR software (Goedhart & Luijsterburg, 2020) (https://huygens.science.uva.nl/VolcaNoseR/). Heat maps were generated using GraphPad Prism 10.1.2. The online app bioRender was used to create a study design schematic presented in Figure 2 (https://www.biorender.com). The Database for Annotation, Visualization, and Integrated Discovery (DAVID, https://davidbioinformatics.nih.gov/) was used to perform the Kyoto Encyclopedia of Gene and Genomes (KEGG) pathway analysis.

## 3 RESULTS

### 3.1 GAA activity is elevated in the liver of diabetic mice

**FIGURE 1:**
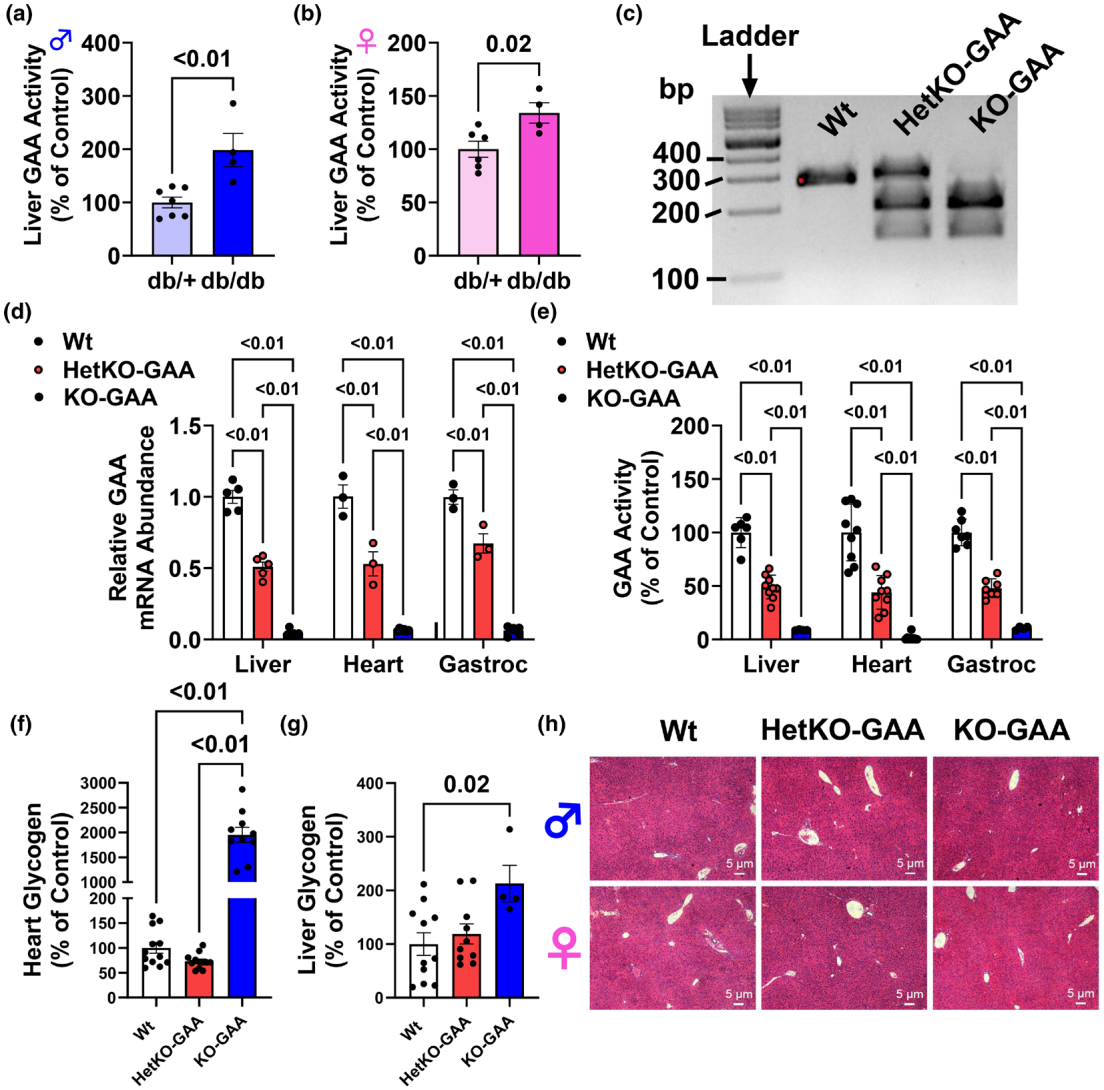
GAA activity is elevated in the liver of db/db mice and characterization of heterozygous and homozygous GAA knockout mice. (a, b) Liver GAA activity in db/+ and db/db male (a) and female (b) mice. (c) Southern blot image of wildtype (Wt), heterozygous knockout of GAA (HetKO‐GAA), and homozygous knockout of GAA (KO‐GAA). (d) Relative GAA mRNA abundance in several mouse tissues from male and female mice. (e) GAA activity in several mouse tissues from male and female mice. (f) Heart glycogen content in male and female mice. (g) Liver glycogen content in male and female mice. (h) Hematoxylin and eosin (H&E) staining of liver sections from male and female mice. Results are expressed as means ± S.E.M.

Given that glycophagy‐derived glucose may contribute to liver glucose output, an upregulation in liver glycophagy may contribute to hyperglycemia in diabetes. To test this idea, GAA activity in the liver of db/+ and db/db diabetic mice was measured. Liver GAA activity was moderately elevated in the liver of both male and female db/db mice compared to db/+ controls (Figure 1a,b), suggesting that, in the setting of diabetes, liver glycophagy is elevated and may contribute to hyperglycemia.

### 3.2 Generation of mice with a heterozygous knockout of GAA

Although liver GAA activity was elevated and associated with diabetes in db/db mice, this is a genetically induced model of obesity and diabetes. Therefore, next we wanted to determine if liver GAA activity was modulated in a HFD induced model of obesity and prediabetes, and if targeting glycophagy could improve glycemic control and other indices of metabolic health. A southern blot was used to genotype and identify Wt, HetKO‐GAA, and homozygous GAA‐KO mice (Figure 1c). To further confirm our mouse model, GAA mRNA abundance (Figure 1d) and GAA activity (Figure 1e) were measured and in Wt control littermates these were significantly greater compared to HetKO‐GAA and KO‐GAA mice across multiple tissues including the liver, heart, and gastrocnemius (gastroc) skeletal muscle. GAA mRNA abundance and activity were also significantly different between HetKO‐GAA and KO‐GAA mice in these tissues. In the heart, KO‐GAA mice had a massive accumulation of glycogen compared to Wt and HetKO‐GAA mice (Figure 1f), which is a classic characteristic of Pompe disease. In the liver, glycogen content was greater in KO‐GAA mice compared to Wt mice, but the magnitude of difference was much less compared to the heart (Figure 1g), while H&E staining of liver sections did not reveal any obvious morphological alterations between genotypes (Figure 1h).

### 3.3 Liver GAA activity, glycogen content, body weight gain, and body composition

**FIGURE 2:**
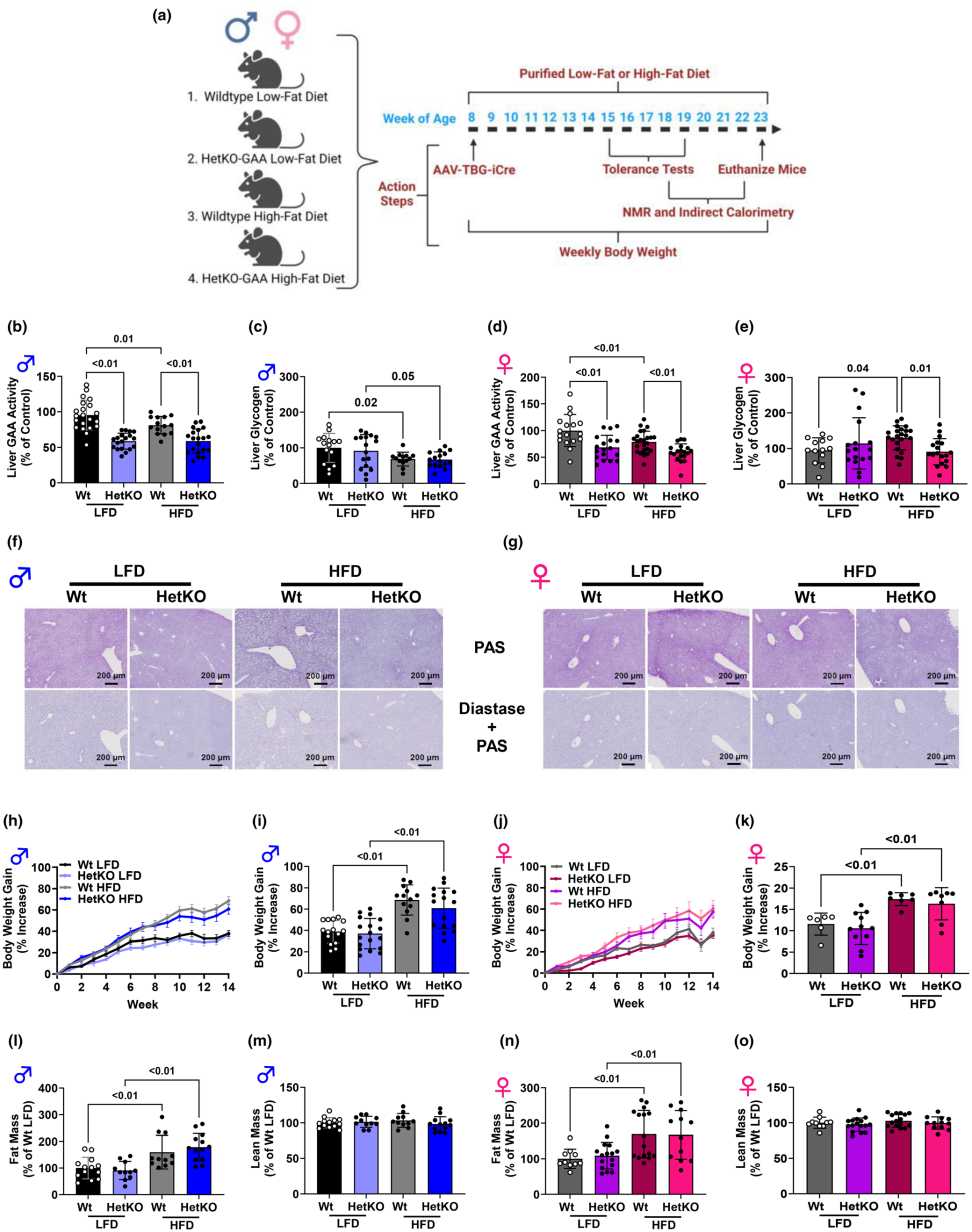
Partially reducing liver GAA activity does not alter liver glycogen or body composition. (a) Schematic showing study design. This figure was created using bioRender software. (b–e) Liver GAA activity (b, d) and glycogen (c, e) in males (b, c) and females (d, e). (f, g) Liver histology with PAS or Diastase + PAS staining in males (f) and females (g). (h–k) Mouse body weight time course (h, j) and total weight gain (i, k) in males (H, I) and females (j, k). (l–o) Mouse body fat (l, n) and lean mass (m, o) as a percentage of total weight in males (l, m), and females (n, o). Results are expressed as means ± S.E.M.

Mice with Pompe disease develop massive amounts of glycogen accumulation in the heart and skeletal muscle that impair muscle function and can induce pathologies such as muscle wasting and heart failure. To avoid these pathology effects, we used HetKO‐GAA mice, which do not develop heart pathology, to determine if partial reductions in glycophagy could alter liver metabolism and overall metabolic health. Approximately 8‐week‐old male and female mice (both Wt and HetKO‐GAA) were placed on a purified LFD or HFD for 15 weeks and then sacrificed (Figure 2a). The HFD reduced GAA activity in both male and female Wt mice, while GAA activity was reduced by approximately 50% in HetKO‐GAA mice compared to Wt mice fed either a LFD or HFD (Figure 2b,d). Glycogen was not different between the groups when measured with a colorimetric assay (Figure 2c,e) or on liver sections with PAS staining (Figure 2f,g). Both male and female mice on the HFD gained more weight (Figure 2h–k) and had a higher fat mass (Figure 2l,n) than mice on the LFD diet, but there was no difference between Wt and HetKO‐GAA mice. Lean mass was not impacted by diet or genotype (Figure 2m,o).

### 3.4 Oxygen consumption and energy expenditure

**FIGURE 3:**
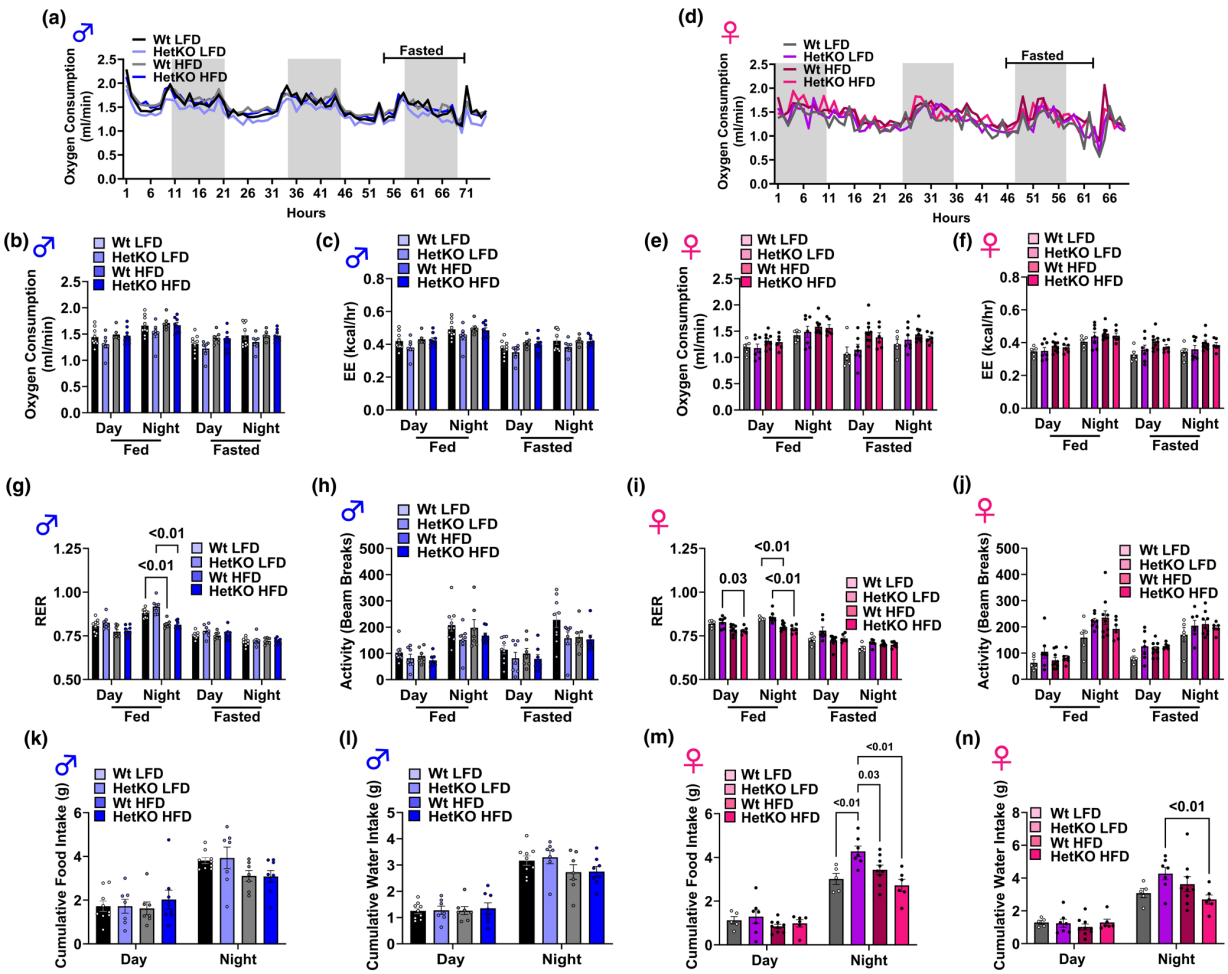
Indirect calorimetry. (a–f) Time course of oxygen consumption (a, d), average oxygen consumption (b, e), and energy expenditure (EE) (c, f) in male (a–c) and female (d–f) mice. (g, i) The respiratory exchange ratio (RER) in male (g) and female (I) mice. (h, j) Physical activity (beam breaks) in male (h) and female (j) mice. (k–n) Cumulative food (k, m) and water intake (l, n) in male (k, l) and female (m, n) mice. Results are expressed as means ± S.E.M.

To assess if diet or genotype impacted whole body oxygen consumption or energy expenditure, mice were housed in the Promethion indirect calorimetry unit for ~70–72 h. In male mice, neither oxygen consumption nor energy expenditure was altered during ad libitum fed conditions or during an overnight fast (Figure 3a–c). Similarly, in females, HetKO‐GAA mice did not have any alteration in oxygen consumption or energy expenditure when fed a LFD or HFD (Figure 3d–f).

### 3.5 Substrate metabolism

Under fed conditions during the night, both Wt and HetKO‐GAA male and female mice fed the HFD had a lower respiratory exchange ratio (RER) compared to HetKO‐GAA male and female mice fed the LFD, indicating greater fat oxidation (Figure 3g,i). However, during fasting conditions, these differences in substrate metabolism disappeared, as the RER was similar across all groups.

### 3.6 Physical activity, food, and water intake

In male and female mice, there was no significant difference in activity (beam breaks) between the groups during the day or night under fed or fasted conditions (Figure 3h,j). During the day, food intake was not different between the groups in both males (Figure 3k) and females (Figure 3m). During the night, food intake between the male groups was not different (Figure 3k), while the HetKO‐GAA female mice fed the LFD had higher food intake compared to the other three groups (Figure 3m). In males, water intake was not different across groups either during the day or night (Figure 3l). In females, water intake during the day was not different across groups, but during the night, water intake was higher in HetKO‐GAA mice fed the LFD compared to HetKO‐GAA mice fed the HFD (Figure 3n).

### 3.7 Glucose, insulin, and glycerol tolerance tests

**FIGURE 4:**
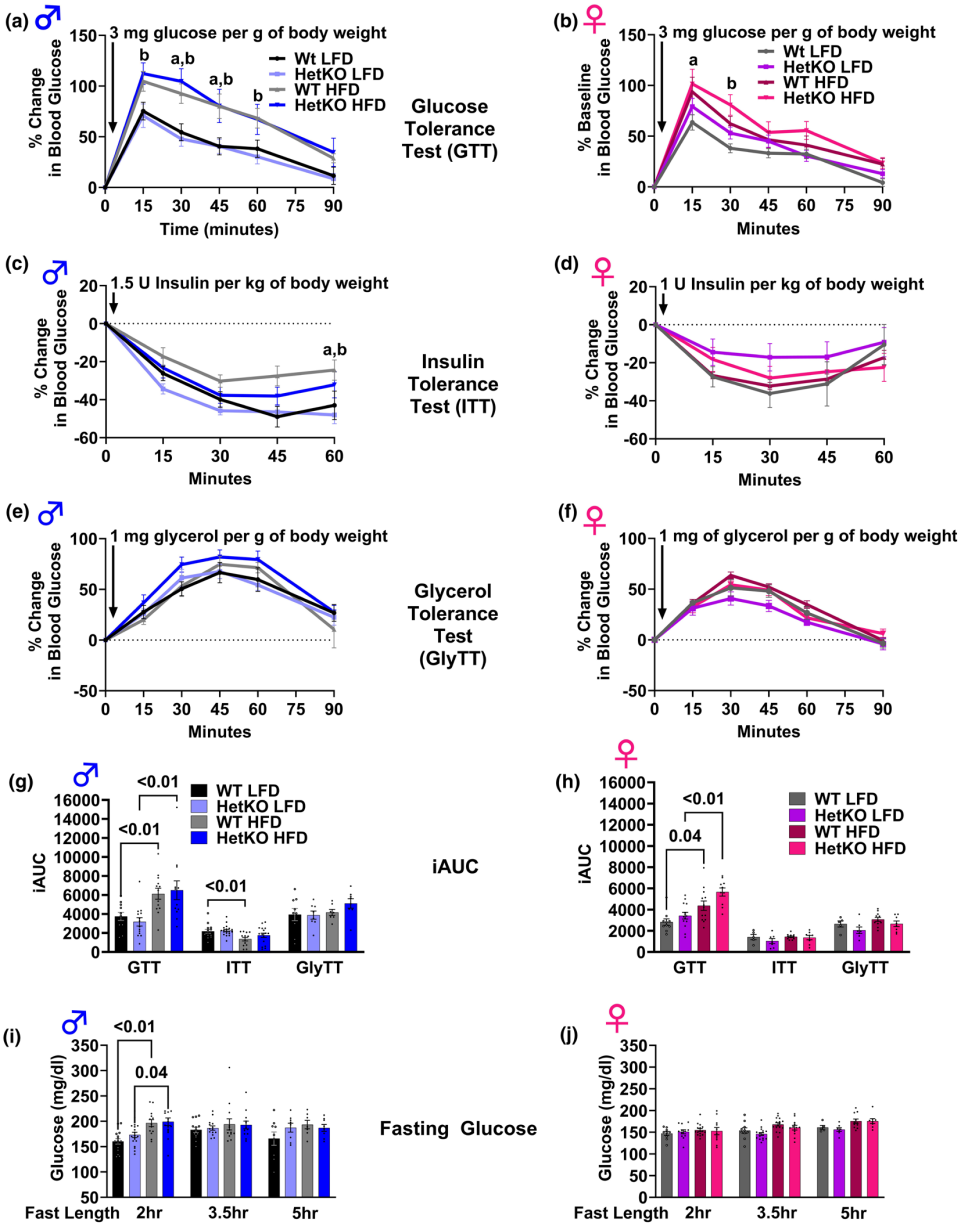
Tolerance tests. (a–f) Glucose tolerance (a, b), insulin tolerance (c, d), and glycerol tolerance (e, f) in male (a, c, e) and female (b, d, f) mice. (g, h) Incremental area under the curve for tolerance tests in males (g) and females (h). (i, j) Fasting blood glucose after various lengths of fasting after ad libitum feeding in male (i) and female (j) mice. Results are expressed as means ± S.E.M.

In both male and female mice, blood glucose concentrations during a glucose tolerance test (Figure 4a,b,g,h) were higher in mice fed the HFD compared to mice fed the LFD, an effect that was not worsened in HetKO‐GAA mice. Fasting blood glucose was higher in male mice fed the HFD, but only after a 2‐h fast, while no other differences in blood glucose were observed after 3.5‐h or 5‐h fasting durations (Figure 4i,j). An insulin tolerance test revealed male mice fed the HFD, regardless of genotype, had higher blood glucose concentrations at 60 min post insulin injection compared to mice fed the LFD, indicating insulin resistance (Figure 4c), although the area under the curve was only significantly different between LFD and HFD Wt groups (Figure 4g). In females, blood glucose concentrations during the insulin tolerance test were not significantly modified between the groups (Figure 4d,h), which is consistent with a previous study showing female mice are protected from HFD‐induced insulin resistance (Pettersson et al., 2012). Glycerol tolerance, an indicator of hepatic gluconeogenesis, was not modified between the groups in male or female mice (Figure 4e–h).

### 3.8 Primary hepatocyte glycogenolysis, glucose output, and insulin action

**FIGURE 5:**
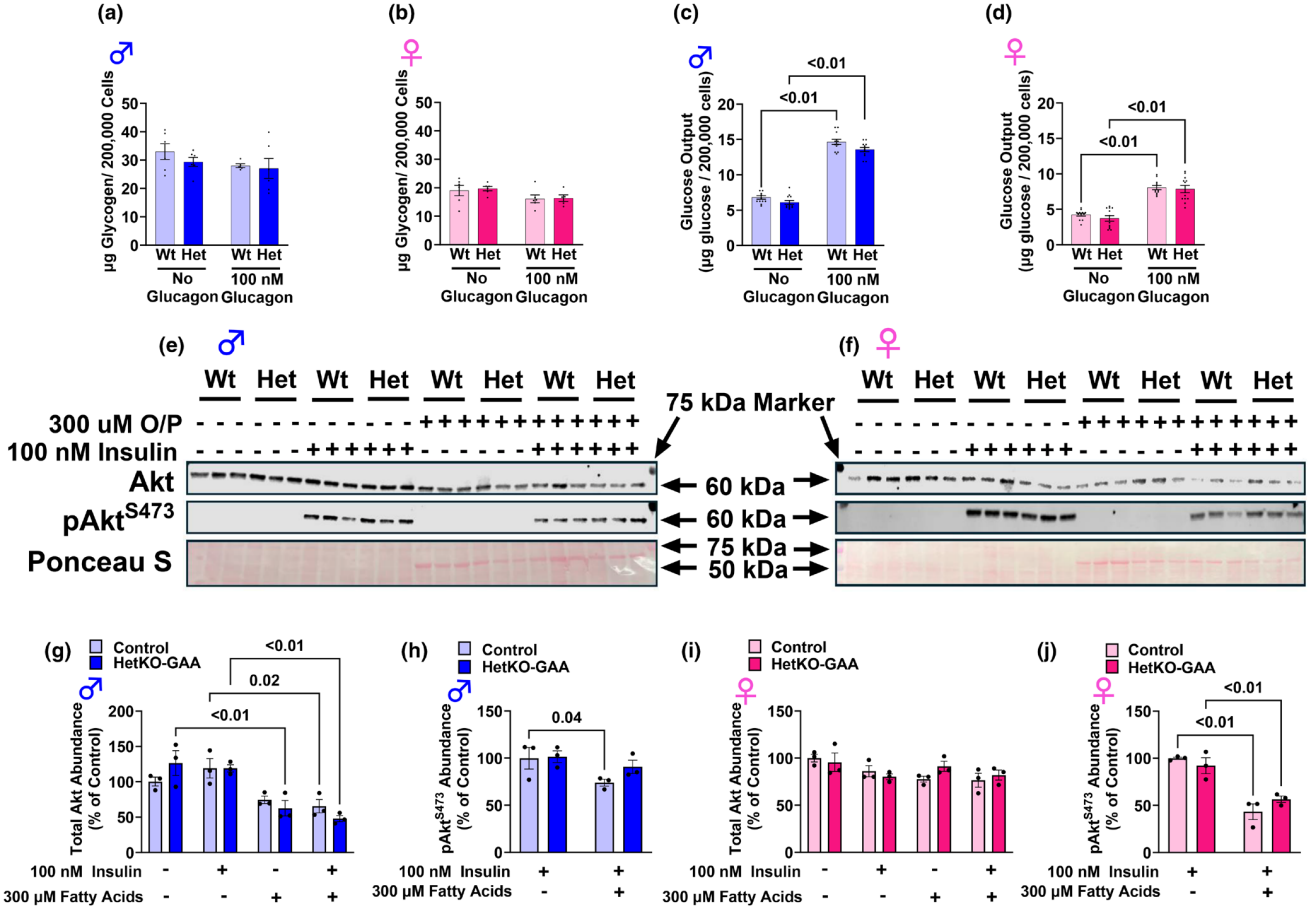
Glycogen, glucose output, and insulin action in primary hepatocytes. (a, b) Glycogen content in primary hepatocytes from male (a) and female (b) mice. (c, d) Glucose output during a 30 min incubation period in primary hepatocytes from male (c) and female (d) mice. (e, h) Western blot image of total Akt and pAkt^S473^ in primary hepatocytes from male (e) and female (h) mice either with or without insulin or oleate/palmitate (O/P) loading. (f–j) Densitometry quantification from Western blot images of total Akt and pAkt^S473^ in primary hepatocytes from male (f, g) and female (i, j) mice either with or without insulin or fatty acid (O/P) loading. Results are expressed as means ± S.E.M.

**FIGURE 6:**
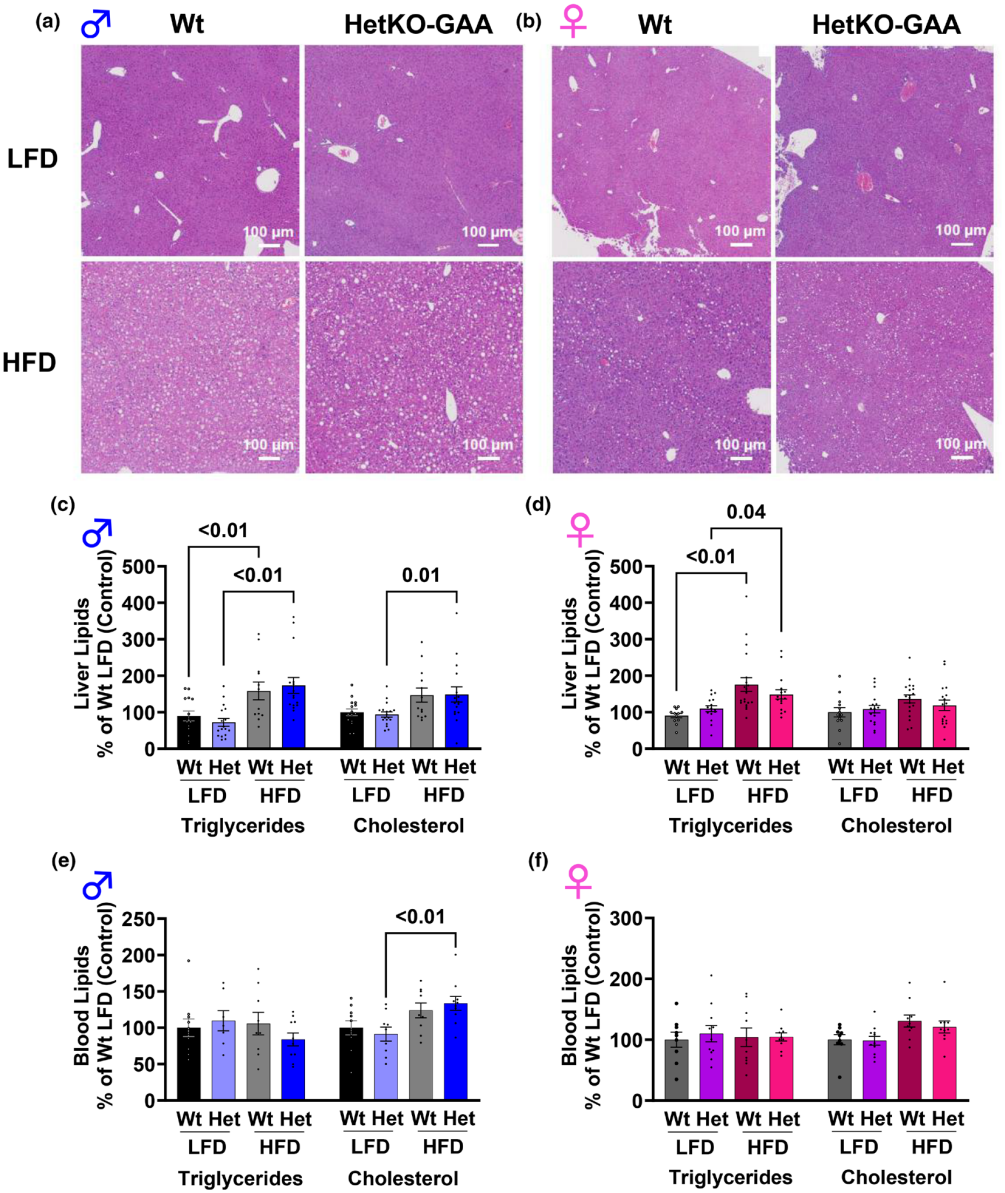
Lipid metabolism. (a, b) Liver hematoxylin and eosin (H&E) staining of liver sections from male (a) and female (b) mice. (c, d) Liver triglyceride and cholesterol levels in male (c) and female (d) mice. (e, f) Blood triglyceride and cholesterol levels in male (e) and female (f) mice. Results are expressed as means ± S.E.M.

Although liver tissue is made up of predominantly hepatocytes, numerous other cells are present in the tissue, including endothelial cells, Kupffer cells, hepatic stellate cells, epithelial cells, and smooth muscle cells. To better understand hepatocyte‐specific metabolism, primary hepatocytes were isolated from male and female mice. Cellular glycogen in male and female mice was not different between groups under basal (non‐glucagon stimulated) or glucagon‐stimulated (100 nM) conditions (Figure 5a,b), which is consistent with our in vivo data suggesting that partial loss of GAA enzyme activity is not sufficient to induce glycogen accumulation. Glucose output in primary hepatocytes significantly increased in the presence of 100 nM glucagon, but this increase was not different between genotypes in hepatocytes from male or female mice (Figures 5c and 6d). We also assessed insulin action in primary hepatocytes that were either incubated in fatty acid‐free media or in media with 300 μM lipids (including 150 μM oleate and 150 μM palmitate). After serum starving cells for ~3 h, with or without lipids, and then stimulating them with 100 nM of insulin for 10 min, the protein abundance of total Akt and phosphorylated (p) Akt^S473^ was assessed. In males, partial depletion of GAA did not alter total Akt or pAkt^S473^ protein abundance in response to insulin stimulation, either without or with lipids, indicating no difference in insulin action between genotypes (Figure 5e–g). Similarly, in primary hepatocytes from female mice, partial GAA deletion did not alter total Akt or pAkt^S473^ protein abundance compared to Wt control hepatocytes (Figure 5h–j). In primary hepatocytes from Wt males and Wt and HetKO females the lipid loading significantly reduced the protein abundance of pAkt^S473^ in response to insulin stimulation, indicating reduced insulin action and increased sensitivity to lipid‐induced insulin resistance. In males, but not females, total Akt protein abundance was significantly reduced with lipid loading (Figure 5f).

### 3.9 Liver morphology and liver and serum triglyceride and cholesterol content

Hematoxylin and eosin staining did not reveal any differences in liver morphology between Wt and HetKO‐GAA mice, although mice fed the HFD, independent of sex and genotype, appeared to have higher lipid droplet accumulation (white circle structures) (Figures 6a,b). Triglycerides and cholesterol were extracted from liver tissue and measured, and this revealed that males, independent of genotype, had higher liver triglyceride content when fed the HFD compared to males fed the LFD (Figure 6c). Liver cholesterol content was higher in the HetKO male mice fed the HFD compared to HetKO mice fed the LFD, while cholesterol was not significantly different between Wt mice fed the LFD and HFD. Both Wt and HetKO female mice fed the LFD had lower liver triglyceride content compared to Wt and HetKO‐GAA mice fed the HFD, while liver cholesterol content was not altered between groups (Figure 6d). In males and females, serum triglycerides were not significantly different between groups (Figure 6e,f). Serum cholesterol was higher in male HetKO‐GAA mice fed the HFD compared to male HetKO‐GAA mice fed the LFD, while cholesterol content was not significantly different between the other groups (Figure 6e,f).

### 3.10 Spatial transcriptomics

**FIGURE 7:**
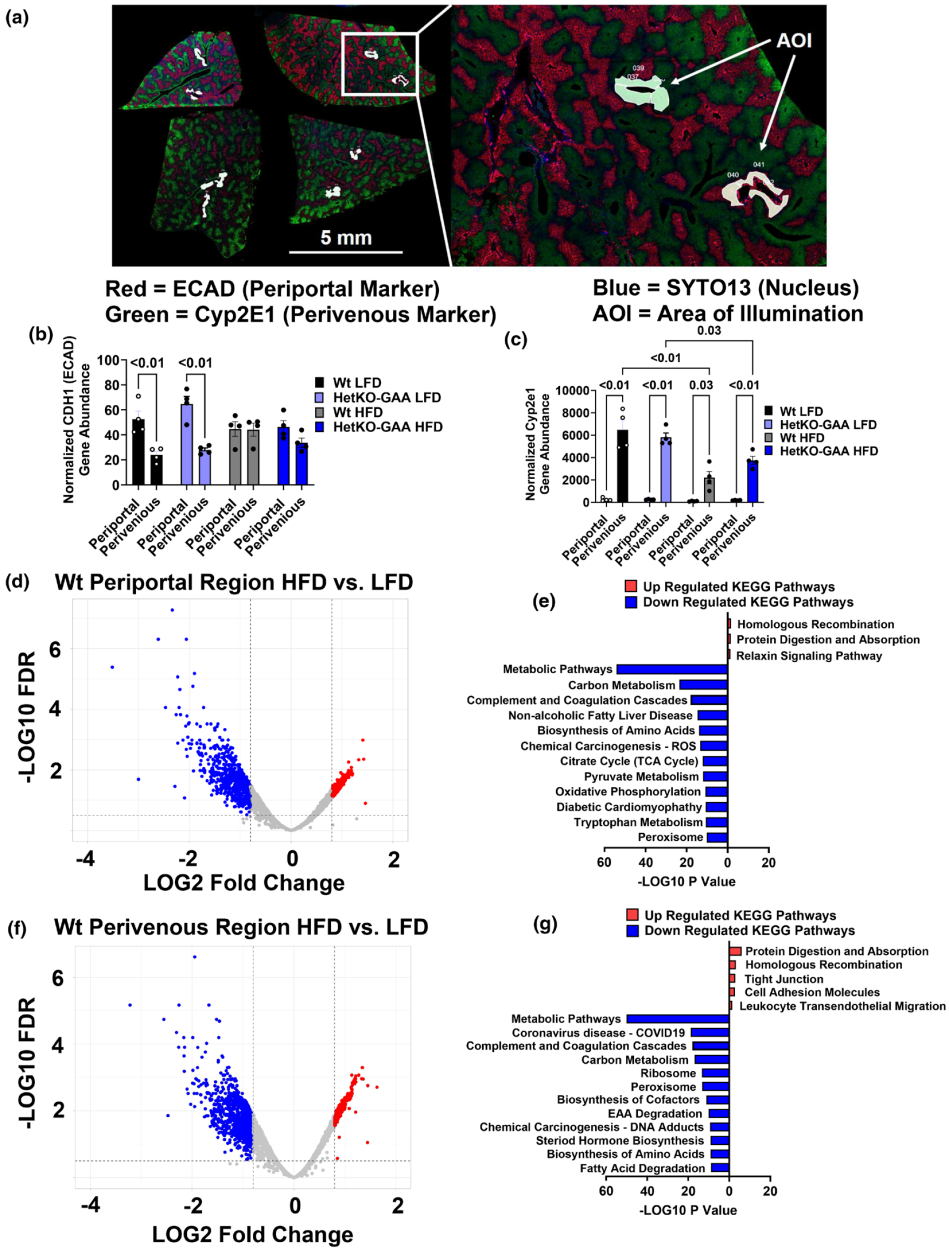
Spatial transcriptomics on male liver sections. (a) Liver sections stained for the periportal marker ECAD (E‐Cadherin) or Cyp2e1 (Cytochrome P450 family 2 subfamily E member 1). (b) Normalized CDH1 (ECAD) gene abundance. (c) Normalized Cyp2e1 gene abundance. (d) Volcano plot showing differentially expressed downregulated genes (blue) or upregulated genes (red) in periportal hepatocytes from HFD fed wildtype control mice. (e) Kyoto Encyclopedia of Gene and Genomes (KEGG) pathways upregulated or downregulated by the HFD in periportal hepatocytes. (f) Volcano plot showing differentially expressed downregulated genes (blue) or upregulated genes (red) in perivenous hepatocytes from HFD fed wildtype control mice. (g) KEGG pathways upregulated or downregulated by the HFD in periportal hepatocytes.

A spatial transcriptomics analysis on male liver sections was performed to determine if genotype or diet impacted liver zone‐specific transcriptomes. The raw data for this analysis was deposited into the Gene Expression Omnibus data repository under series record GSE284331 and is publicly available. Periportal hepatocytes were stained red with an antibody against the ECAD protein (a known marker of the periportal zone), perivenous hepatocytes were stained green with an antibody against Cyp2E1 (a known marker for perivenous hepatocytes), and the nucleus was stained blue with an antibody against SYTO13 (Figure 7a). We then used these markers to create three AOI's within the periportal and perivenous zone that was used for RNA sequencing (Figure 7a). We then averaged the three AOI's within each zone and used the average for data analysis (All normalized data used in analysis are available in Tables S1 and S2). A total of 7395 genes were detected. We confirmed that each zone was correctly identified using the gene expression of CDH1 (ECAD) and Cyp2E1 in each zone. In all mice fed the LFD, Cdh1 gene expression was higher in periportal compared to perivenous hepatocytes, while in HFD‐fed mice this difference in mRNA abundance between zones disappeared (Figure 7b). Cyp2e1 gene abundance was higher in perivenous hepatocytes compared to periportal hepatocytes in all mice, independent of genotype (Figure 7c). However, Cyp2e1 abundance was lower in perivenous hepatocytes in mice fed the HFD compared to mice fed the LFD (Figure 7c). Our initial analysis compared periportal and perivenous transcriptomes between Wt control and HetKO‐GAA mice (Tables S3–S6). When comparisons were made between Wt and HetKO‐GAA periportal or perivenous zones in male mice fed the LFD, no significant differences were observed (Tables S3 and S4). When comparisons were made between Wt and HetKO‐GAA periportal hepatocytes from mice fed the HFD, only 6 genes were significantly different that met our criteria, while in the perivenous zone only 4 genes were significantly different (Tables S5 and S6).

**FIGURE 8:**
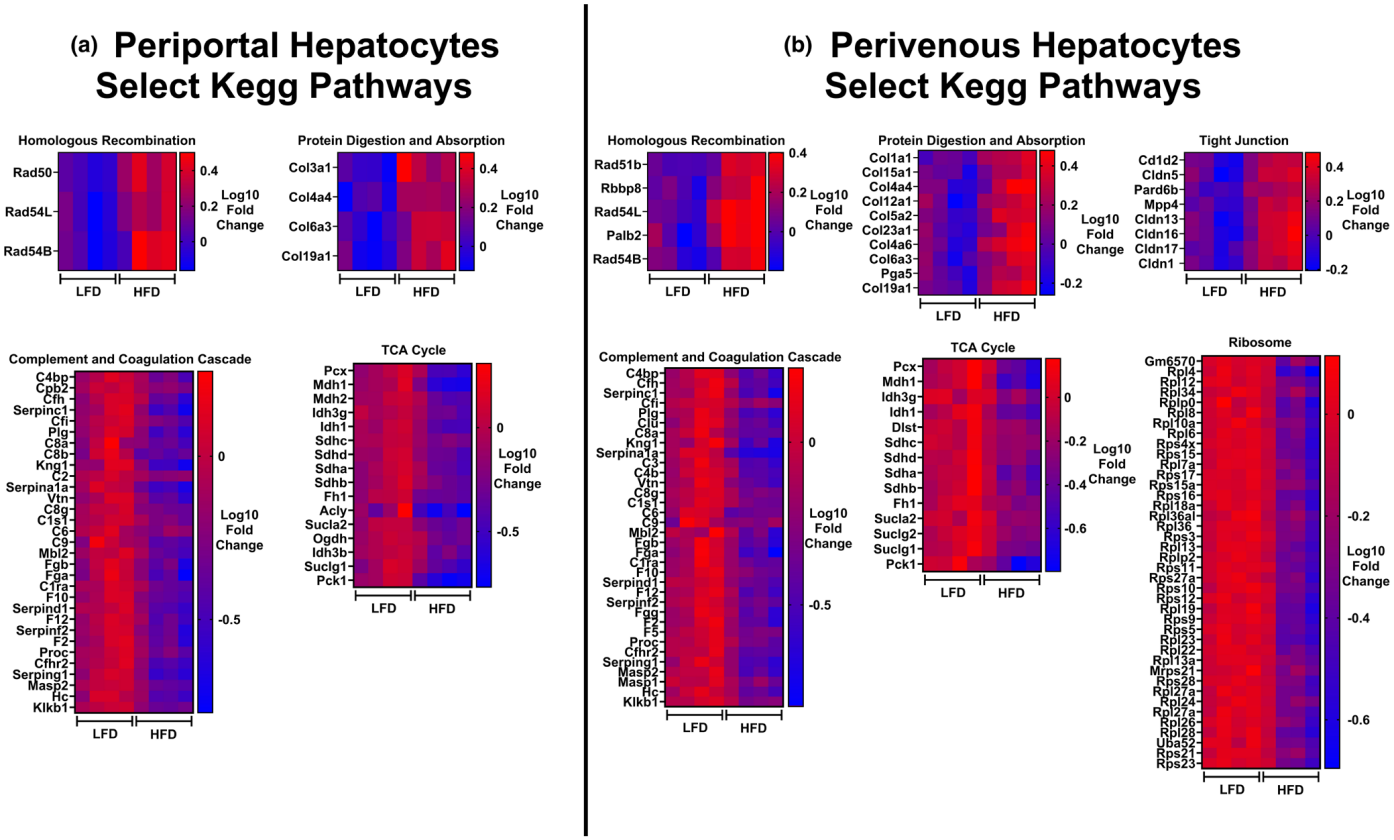
Heatmaps showing select genes and KEGG pathways upregulated or downregulated by the HFD in periportal and perivenous hepatocytes. (a) Heatmaps showing select genes and KEGG pathways in periportal hepatocytes that were differentially modified by the HFD. (b) Heatmaps showing select genes and KEGG pathways in perivenous hepatocytes that were differentially modified by the HFD.

Since HetKO‐GAA did not have a significant impact on gene abundance, we compared periportal and perivenous hepatocytes between Wt LFD‐fed and Wt HFD‐fed mice to examine how diet impacts periportal and perivenous hepatocyte gene abundance (All data available in Tables S7 and S8). In the periportal region, the HFD increased the abundance of 161 genes and decreased the abundance of 578 genes (Figure 7d). A KEGG pathway analysis revealed that periportal genes involved in homologous recombination, protein digestion and absorption, and relaxin signaling were increased by the HFD, while the abundance of genes involved in metabolic pathways was reduced by the HFD, including pathways related to carbon metabolism, biosynthesis of amino acids, TCA cycle, pyruvate metabolism, and oxidative phosphorylation, to name a few, suggesting a metabolic signature of reduced mitochondrial metabolism in periportal hepatocytes (Figures 7e and 8a), a region that typically has higher rates of mitochondrial metabolism compared to perivenous hepatocytes.

In the perivenous region, the HFD increased the abundance of 297 genes and decreased the abundance of 736 genes (Figure 7f), indicating that the HFD has a bigger impact on more genes in the perivenous region compared to the periportal region. A KEGG pathway analysis revealed that although there was some overlap between periportal and perivenous zones in changes in gene abundance due to the HFD, there were also some notable differences (Figure 7g). For instance, in perivenous hepatocytes, the HFD increased genes involved in the KEGG pathways tight junction, cell adhesion, and leukocyte transendothelial migration, whereas these genes were not altered in the periportal hepatocytes. Many of the same KEGG metabolic pathways were downregulated in perivenous hepatocytes that were downregulated in periportal hepatocytes, with the exception that ribosome‐associated genes were significantly impacted by the HFD in perivenous hepatocytes, but not periportal hepatocytes (Figure 8b).

## 4 DISCUSSION

We examined how HetKO‐GAA impacts metabolic health (i.e., obesity, insulin sensitivity, glucose tolerance, fatty liver) and liver metabolism (i.e., liver glycogen content, liver glucose output, substrate metabolism, and energy expenditure) in a mouse model of HFD‐induced obesity and prediabetes. Our results indicate that partial heterozygous disruption of the GAA gene is nonconsequential to several indices of liver metabolism and overall metabolic health. These data suggest that hepatocytes produce more than enough GAA enzyme than is required for adequate glycophagy flux, as glycogen did not accumulate in the liver of mice with partial genetic silencing of GAA, nor did it impact metabolism.

Data from previous studies have suggested that the metabolic fate of glucose liberated via glycophagy in the liver is glucose output (Douillard‐Guilloux et al., 2010; Hijmans et al., 2017). In the setting of obesity, insulin resistance, prediabetes, and diabetes, a dysregulated increase in liver glucose output contributes to hyperglycemia, and thus targeting liver glycophagy seems logical to reduce liver glucose output in this context. We show that db/db mice, which have a spontaneous mutation in the leptin receptor that promotes obesity and type 2 diabetes, had reduced liver GAA activity. However, in a HFD induced obesity and prediabetes model, HetKO‐GAA is not sufficient to reduce liver glucose output and has no impact to alter glucose tolerance, suggesting that partial disruption of the GAA gene is insufficient to alter liver metabolism. Inhibiting GAA activity with the FDA‐approved drugs acarbose or miglitol improves glucose tolerance and insulin action (Goda et al., 2007; Joubert et al., 1990; Komatsu et al., 2018; Nozaki et al., 2009), and the mechanism of action is thought to primarily occur in the intestine by intestinal GAA inhibition since these drugs are taken orally (Ahr et al., 1989; Joubert et al., 1990). However, miglitol, unlike acarbose, has some bioavailability; thus, it may reach peripheral tissues (Ahr et al., 1997) and has many positive effects in the setting of obesity and diabetes, including increasing glycemic control (Goda et al., 2007), energy expenditure (Sugimoto et al., 2014), and reducing obesity and fatty liver disease (Kishida et al., 2017; Komatsu et al., 2018), possibly due to effects of GAA inhibition in peripheral tissues, although this is not fully established. Given that miglitol is very potent at inhibiting GAA activity, it is possible that to achieve metabolic health effects in the liver, a complete inhibition or knockout of GAA may be required. In support of this, incubating hepatocytes directly with acarbose, another potent GAA inhibitor, was able to reduce glucose output, suggesting a complete inhibition of GAA is required for metabolic effects to occur (Hijmans et al., 2017).

The liver is essential for metabolic homeostasis as well as the detoxification and elimination of various xenobiotic and toxic compounds (Trefts et al., 2017). Across the liver lobule are spatially zonated hepatocytes which allow the liver to deal with this range of tasks (Aizarani et al., 2019; Braeuning et al., 2006; Yu et al., 2022). In general, the liver can be divided into two zones, including the periportal zone, which receives nutrient‐rich blood from the intestine and is more primed for oxidative metabolism, amino acid degradation, and gluconeogenesis, and the perivenous zone, which receives nutrient‐poor blood and is more primed for glycolysis, bile acid synthesis, and xenobiotic metabolism (Aizarani et al., 2019; Braeuning et al., 2006; Yu et al., 2022). To our knowledge, this is the first study to use spatial transcriptomics to examine changes in gene abundance in periportal and perivenous hepatocytes between LFD‐fed and HFD‐fed male mice. The spatial transcriptomics analysis on liver sections afforded insight into zone‐specific effects of both genotype and diet on gene expression. Although HetGAA KO did not have a robust effect on gene expression in either zone, HFD feeding had a robust impact on gene abundance in both zones, although more genes were significantly impacted in the perivenous zone (1033 total genes significantly different) compared to the periportal zone (739 total genes significantly different). Other reports have performed whole liver RNA sequencing in mice in response to a HFD with samples containing RNA from both periportal and perivenous hepatocytes (Ding et al., 2023; Soltis et al., 2017). In general, these studies show that the HFD alters the abundance of genes involved in metabolic processes such as lipid or amino acid metabolism. However, a direct comparison to our findings is difficult due to discrepancies in the diets used. For instance, Ding et al. (Ding et al., 2023) compared chow‐fed mice to purified HFD‐fed mice (D12109C, Research Diets), and this design resulted in 6078 differentially expressed genes in the liver, whereas we found much fewer differentially expressed genes when comparing a purified LFD (TD.180916, Inotiv) to a purified HFD (TD.06415, Inotiv). Similarly, Soltis et al. (Soltis et al., 2017) compared a chow diet to a HFD (S3282, Bioserve) that is not identical to the diet we fed the mice.

A unique finding of our spatial transcriptomics data set was that our KEGG pathway analysis revealed ribosome‐related gene abundance was reduced in perivenous hepatocytes, but not periportal hepatocytes, in obese, HFD‐fed mice. Ribosomes perform protein synthesis within the cell, and despite obesity being characterized as a condition of over‐nutrition which would be expected to increase protein synthesis, protein synthesis in this setting has been shown to be downregulated in the liver of obese mice (Fu et al., 2012). Our findings extend upon these past findings and suggest this impairment may occur exclusively in perivenous hepatocytes, although it is not clear why. Given that perivenous hepatocytes generally have higher rates of protein synthesis, they may be more sensitive to HFD‐ and obesity‐induced reductions in protein synthesis compared to periportal hepatocytes, which generally have lower rates of protein synthesis and increased amino acid metabolism to support gluconeogenesis (Braeuning et al., 2006; Jungermann, 1986).

In summary, partial inhibition of GAA is nonconsequential to liver metabolism or metabolic health in male or female mice with HFD‐induced obesity and prediabetes, suggesting that the liver produces more than enough GAA enzyme for adequate glycophagy flux. Whether a complete inhibition of liver GAA is required for metabolic health effects should be assessed in future studies. HFD feeding robustly reduces genes involved in metabolism in periportal and perivenous hepatocytes, while uniquely reducing ribosome‐related genes in perivenous hepatocytes only. Future investigations are required to determine why perivenous hepatocytes, but not periportal hepatocytes, experience a reduction in ribosome gene abundance in response to HFD‐induced obesity and prediabetes. Furthermore, the unique transcriptome in periportal and perivenous hepatocytes from HFD‐induced obese mice highlights novel targets that could be exploited to improve liver function and metabolic health in obesity.

## AUTHOR CONTRIBUTIONS

Cameron P. McCall: performed experiments, analyzed data, interpreted results of experiments, prepared figures, drafted manuscript, edited and revised manuscript, approved final version of themanuscript. Melina C. Mancini: performed experiments, edited and revised the manuscript, approved the final version of themanuscript. Jaroslaw Staszkiewicz: analyzed data, prepared figures, edited and revised the manuscript, approved the final version of themanuscript. Douglas G. Mashek: Conceived and designed research, edited and revised the manuscript, approved the final version of themanuscript. Timothy D. Heden: Conceived and designed research, performed experiments, analyzed data, interpreted results of experiments, prepared figures, drafted manuscript, edited and revised manuscript, and approved final version of manuscript.

## FUNDING INFORMATION

Research reported in this publication was supported by the National Institute of General Medical Sciences under award number R35GM154665 (awarded to TDH), the National Institute of Diabetes, Digestive, and Kidney Diseases under award number K01DK125258 (awarded to TDH) of the National Institutes of Health, and the National Institute of General Medical Sciences under award number P20GM135002:5359 (subproject awarded to TDH, principal investigator was Jacqueline M Stephens). This work was supported in part by the Spatialomics services provided by the resources and staff at the University of Minnesota University Imaging Centers.

## CONFLICT OF INTEREST STATEMENT

The authors have no conflicts of interest to disclose.

## DISCLAIMERS

The authors have no disclaimers to report.

## ETHICS STATEMENT

All animal experiments were approved by the Institutional Animal Care and Use Committee at the University of Minneosta or Pennington Biomedical Research Center.

## Supporting information


Table S1.

## Data Availability

The raw data for spatial transcriptomics was deposited into the Gene Expression Omnibus data repository under series record GSE284331 and is publicly available. Source data for spatial transcriptomics is available in Tables S1 and S2. All other source data is available from the corresponding author upon request.
